# Cell-based non-invasive prenatal testing for monogenic disorders: confirmation of unaffected fetuses following preimplantation genetic testing

**DOI:** 10.1007/s10815-021-02104-5

**Published:** 2021-03-07

**Authors:** Christian Liebst Frisk Toft, Hans Jakob Ingerslev, Ulrik Schiøler Kesmodel, Lotte Hatt, Ripudaman Singh, Katarina Ravn, Bolette Hestbek Nicolaisen, Inga Baasch Christensen, Mathias Kølvraa, Line Dahl Jeppesen, Palle Schelde, Ida Vogel, Niels Uldbjerg, Richard Farlie, Steffen Sommer, Marianne Louise Vang Østergård, Ann Nygaard Jensen, Helle Mogensen, Kristín Rós Kjartansdóttir, Birte Degn, Henrik Okkels, Anja Ernst, Inge Søkilde Pedersen

**Affiliations:** 1 Department of Molecular Diagnostics, Aalborg University Hospital, Aalborg, Denmark; 2 Department of Clinical Medicine, Aalborg University, Aalborg, Denmark; 3 Fertility Unit, Aalborg University Hospital, Aalborg, Denmark; 4 ARCEDI Biotech ApS, Vejle, Denmark; 5 Department of Clinical Genetic, Aarhus University Hospital, Aarhus, Denmark; 6 Department of Obstetrics and Gynecology, Aarhus University Hospital, Aarhus, Denmark; 7 Department of Obstetrics and Gynecology, Viborg Regional Hospital, Viborg, Denmark; 8 Department of Obstetrics and Gynecology, Horsens Regional Hospital, Horsens, Denmark; 9 Department of Obstetrics and Gynecology, Randers Regional Hospital, Randers, Denmark; 10 Department of Obstetrics and Gynecology, Aalborg University Hospital, Aalborg, Denmark; 11 Department of Obstetrics and Gynecology, Kolding Regional Hospital, Kolding, Denmark; 12 Molecular Genetics Laboratory, Department of Clinical Genetics, University Hospital Copenhagen, Copenhagen, Denmark

**Keywords:** cbNIPT, PGT-M, Prenatal testing, STR markers

## Abstract

**Purpose:**

Proof of concept of the use of cell-based non-invasive prenatal testing (cbNIPT) as an alternative to chorionic villus sampling (CVS) following preimplantation genetic testing for monogenic disorders (PGT-M).

**Method:**

PGT-M was performed by combined testing of short tandem repeat (STR) markers and direct mutation detection, followed by transfer of an unaffected embryo. Patients who opted for follow-up of PGT-M by CVS had blood sampled, from which potential fetal extravillous throphoblast cells were isolated. The cell origin and mutational status were determined by combined testing of STR markers and direct mutation detection using the same setup as during PGT. The cbNIPT results with respect to the mutational status were compared to those of genetic testing of the CVS.

**Results:**

Eight patients had blood collected between gestational weeks 10 and 13, from which 33 potential fetal cell samples were isolated. Twenty-seven out of 33 isolated cell samples were successfully tested (82%), of which 24 were of fetal origin (89%). This corresponds to a median of 2.5 successfully tested fetal cell samples per case (range 1–6). All fetal cell samples had a genetic profile identical to that of the transferred embryo confirming a pregnancy with an unaffected fetus, in accordance with the CVS results.

**Conclusion:**

These findings show that although measures are needed to enhance the test success rate and the number of cells identified, cbNIPT is a promising alternative to CVS.

**Trial registration number:**

N-20180001

**Supplementary Information:**

The online version contains supplementary material available at 10.1007/s10815-021-02104-5.

## Introduction

Preimplantation genetic testing (PGT) allows for the selection and transfer of unaffected embryos from patients carrying or affected by a hereditary disorder. The most recent report from the European Society of Human Reproduction and Embryology (ESHRE) PGT consortium states that PGT is associated with a low risk of misdiagnosis [1]. Misdiagnosis was reported in 13/12790 (0.1%) of PCR-based cycles and in 21/40640 (0.05%) of FISH cycles, while no data was available from genome-wide analysis.

Despite the low risk, a misdiagnosis has significant consequences for both the couple and the child in question. Hence, prenatal testing, with the aim of verifying the PGT result to allow for termination of pregnancy in case of a misdiagnosis, is recommended by the ESHRE consortium guidelines [2]. Prenatal testing can be performed on feto-placental material obtained by either chorionic villus sampling (CVS) or by amniocentesis, of which the former is preferred as it can be performed earlier in the pregnancy. Hence, the current gold standard for prenatal testing following PGT is CVS, which is an invasive procedure associated with a small risk (< 0.2%) of miscarriage [3]. In our setting, approximately 50% of patients undergoing PGT choose not to opt for prenatal follow-up by CVS despite recommendations and counselling on the (low) risk of misdiagnosis associated with PGT. The reason(s) for declining CVS are unknown, but they likely include factors such as fear of the procedure, risk of miscarriage, and unwillingness to opt for termination of pregnancy in the case of an affected fetus. A non-invasive procedure could be a relevant alternative for the group of patients who opt out of CVS due to the risk and discomfort associated with CVS. In general, replacing invasive procedures with a non-invasive solution for all patients is desirable. However, sufficient validation is required. In a survey among English cystic fibrosis patients, Hill et al. found that while only 43.5% were willing to undergo prenatal invasive testing, 94.4% would choose non-invasive prenatal diagnosis (NIPD) given the opportunity [4]. Eliminating the risk of miscarriage is an important aspect when patients consider prenatal testing in many countries [5], including Denmark [6]. Interestingly, some patients even seem willing to compromise test quality, such as accuracy and genetic information obtained, in order to avoid the risk of miscarriage, as evident from questionnaires among English [7] and Danish patients [6]. Hence, a non-invasive alternative to prenatal follow-up is likely to increase patient acceptance of confirmatory prenatal testing.

Non-invasive prenatal testing (NIPT) of monogenic disorders using cell-free fetal DNA (cffDNA) was demonstrated in 2000 and is currently part of the public health service, as well as being commercially available, in England [8]. CffDNA are fragmented fetal DNA of approximately 150–200 base pairs present in the blood of pregnant women [9–11]. The fragmented state of the DNA poses a challenge during genetic analysis, in particular the analysis of specific genetic defects, such as repeat expansions, that are not detectable using cffDNA. The cffDNA fraction within maternal blood depends on gestational age [12–14] and maternal weight [14–16], sometimes causing problems when performing NIPT during early pregnancy and in overweight patients. Additionally, the inherent excess of maternal cell-free DNA might complicate the diagnosis of maternally inherited monogenetic disorders.

Some of the challenges associated with using cffDNA for NIPT can be avoided when performing cell-based NIPT (cbNIPT). Intact fetal cells provide access to an intact genome for testing, thereby avoiding the issues associated with using fragmented cffDNA. Fetal cells have been successfully isolated from gestational weeks 5 [17] and 6 [18], making cbNIPT possible in early pregnancy. Additionally, isolation of fetal cells is not affected by maternal weight [19]. While the presence of an entire fetal genome in theory allows for testing of all types of genetic defects, the challenge of cbNIPT is to discriminate between fetal and maternal cells and efficient isolation of the former.

Based on gene expression analyses, ARCEDI Biotech has classified circulating fetal cells as placental cells [20], specifically extravillous trophoblasts [21]. A protocol for the enrichment of fetal cells from the blood samples of pregnant women was published by ARCEDI Biotech [22], in which they reported an average output of 12.8 fetal cells from 30 ml of blood (0.43 fetal cells/ml). Most importantly, fetal cells were identified in all samples from 111 pregnancies. The isolated fetal cells proved suitable for downstream analysis by both array comparative genomic hybridization and next-generation sequencing. Another group has reported the isolation of 4.17 trophoblastic cells per 30 ml blood, identifying fetal cells in 102 of 125 blood samples (82%) [23]. Numerous commercial solutions related to single-cell collection and analysis have become available in the last few years [24], but peer-reviewed published proof of their ability and efficacy to identify fetal cells from maternal blood is lacking, likely contributing to the fact that cbNIPT is still awaiting widespread clinical implementation.

To date, cbNIPT has been used to test for the carrier status of monogenic disorders [17, 25, 26] and for aneuploidy, unbalanced structural translocations and smaller deletions [23, 27]. To our knowledge, only one previous publication has reported the use of cbNIPT following PGT-M, presenting data from two couples at risk of transmitting congenital deafness and ichthyosis caused by point mutations or smaller deletions [28]. Here, we present our results from cbNIPT on eight singleton pregnancies following PGT-M for single nucleotide variants, small and large deletions, duplications, and repeat expansions, demonstrating the variety of genetic defects that can be tested in the presented cbNIPT setup. We present a cbNIPT analysis identical to that used for PGT-M based on STR marker analysis and direct mutation detection, thereby enhancing diagnostic accuracy while simultaneously allowing determination of the origin (maternal or fetal) of each analyzed cell sample.

## Materials and methods

### Study population

Between November 2018 and August 2019, eight patients referred to PGT for monogenic disorders at the Fertility Unit at Aalborg University Hospital were recruited. All included patients signed written consent to participate in the study after receiving both written and oral information. The project and patient hand-out information were approved by the North Denmark Region Committee on Health and Research Ethics (Reference: N20180001). Screening for aneuploidy was not performed in any of the eight cases.

### In vitro fertilization, embryo culture, biopsy, and transfer

A detailed description of the in vitro fertilization, embryo culture, biopsy, and transfer is provided in the supplementary. Trophectoderm biopsy and single-embryo transfer of an unaffected embryo were performed in all cases. Pregnancy was defined as the presence of a fetal heartbeat monitored by ultrasound in gestational week 7.

### Preimplantation genetic testing for monogenic disorders

#### Preclinical setup

PGT-M was performed by STR marker analysis, coupled with direct mutation detection when possible. The preclinical setup involves testing the couple prior to PGT to (1) identify suitable STR markers close to the gene of interest and (2) ensure the capability of the direct mutation detection to differentiate between wildtype (unaffected partner) and mutant (affected partner). This obviates the need for positive controls in the traditional sense during PGT and later cbNIPT.

#### STR markers

STR markers are intronic polymorphisms occurring frequently in the human genome. They consist of a short nucleotide sequence tandemly repeated a variable number of times, which makes them effective for distinguishing alleles within and between individuals. During PGT, STR markers serve to track which alleles are inherited by the embryo and thereby allow the distinction of unaffected and affected embryos. Simultaneously, STR markers allow for the detection of DNA contamination, which is especially important in cases with low quantities of input material as is the case in PGT, where 5–10 trophectoderm cells are tested.

For each case, a set of approximately 10 STR markers, preferably 5 on each side of the gene of interest, were identified through the UCSC genome browser (http://genome.ucsc.edu/) and tested on DNA purified from the blood of both parents and relevant relatives in order to phase the alleles and identify informative STR markers. STR markers already annotated in the UCSC genome browser were referred to by their respective locus identifier (e.g., D1S1656), while newly identified STR markers were given a locus name adopted from the name of the gene (Table 1 and S1). All STR markers used, including primer sequences, can be found in the supplementary materials and methods in Table S1.

**Table 1:** Case characteristics

Case number	Affected parent	Age at time of gamete retrieval		Genetic disorder	Gene	Mutation	STR markers used for embryo testing	Direct mutation detection	Blood sampling time (gestational week)
		Maternal	Paternal						
Case 1	Maternal	29	32	Neurofibromatosis type I	*NF1*	c.7907+4_7907+7delAGTA	STR 1 (NF1-4): fully informative (1.7 Mb upstream) STR 2 (D17S1880): semi informative (1.3 Mb downstream)	Yes	10+5
Case 2	Paternal	36	33	Spastic paraplegia type 4	*SPG4*	c.481delG	STR 1 (D2S2255): fully informative (1.1 Mb upstream) STR 2 (SPG4-2): fully informative (1.1 Mb downstream)	Yes	11+4
Case 3	Paternal	29	29	Charcot-Marie-Tooth type A	*PMP22*	(p11.2p12)dup(17)	STR 1 (D17S122): fully informative (0.9 Mb upstream) STR 2 (D17S900): semi informative (inside duplication)	Indirect by STR	11+4
Case 4	Maternal	30	31	CADASIL	*NOTCH3*	c.520T>C	STR 1(D19S892): semi informative (0.7 Mb upstream) STR 2 (D19S252): semi informative (0.4 Mb downstream)	Yes	10+6
Case 5	Maternal	26	32	Fragile X syndrome	*FMR1*	64 CGG-repeats (premutation)	STR 1 (DXS1193): fully informative (1.3 Mb downstream) STR 2 (DXS8086): fully informative (2.3 Mb downstream)	Yes	12+0
Case 6	Maternal	28	29	Duchenne and Becker muscular dystrophy	*DMD*	Deletion of exon 47 and 48	STR 1 (DXS8039): semi informative (0.9 Mb upstream) STR 2 (DXS997): fully informative (Inside deletion) STR 3 (DMD67): fully informative (1.8 Mb downstream)	Indirect by STR	11+0
Case 7	Maternal	34	39	Myotonic dystrophy type 1	*DMPK*	>80 CTG repeats	STR 1 (D19S538): fully informative (1.9 Mb upstream) STR 2 (D19S545): fully informative (1.2 Mb downstream)	Not possible^a^	12+2
Case 8	Maternal	34	35	Marfan syndrome	*FBN1*	c.1148-2A>G	STR 1 (D15S978): fully informative (0.3 Mb downstream)	Yes	13+0

^a^Unaffected maternal allele identical to both paternal alleles

*CADASIL*, cerebral autosomal dominant arteriopathy with subcortical infarcts and leukoencephalopathy; *STR*, short tandem repeat

STR markers were defined as informative if the four alleles of the two parents all differed from each other. STR markers were defined as semi-informative if one or two alleles from the unaffected parent were identical to one of the alleles of the affected parent. In cases where only semi-informative STR markers were available at a locus, STR markers informative for the unaffected allele were prioritized, as diagnosing embryos as unaffected by the presence of the unaffected allele is preferable to basing the diagnosis on the absence of the affected allele (as this can also be caused by allele dropout (ADO)).

Preferably, at least one informative STR marker on each side of the affected gene was identified to minimize the risk of crossover in cases where direct mutation detection was not possible or failed. In some cases, only semi-informative STR markers were available.

Detailed information on preimplantation genetic testing is provided in the supplementary materials and methods section.

#### Data analysis

In data analysis, “no signal” indicates a PCR reaction where no PCR product was detected while “ADO” indicates that the PCR reaction was successful but that one or more alleles expected to be present were absent (allele dropout). The ADO rate was calculated as the number of observed alleles divided by the number of expected alleles:

$$ ADO=\frac{Alleles\ observed}{Alleles\ expected} $$

The ADO rate was reported with a 95% exact confidence interval (Clopper-Pearson).

### cbNIPT

#### Blood sampling and handling

Blood samples were scheduled to be obtained at the day of CVS. In one case (case 8), the blood sample was obtained on a different day (8 days after CVS) due to logistic issues. Thirty milliliters of blood was obtained per patient in Cell-Free DNA BCT® (Streck, Omaha, USA) blood sampling tubes, which were stored at room temperature until collected on site by ARCEDI Biotech within 48 h. The blood sample was processed as previously described with minor modifications [29]. In brief, whole blood was fixed in formaldehyde in phosphate-buffered saline (PBS) with gentle mixing followed by red blood cell lysis in Triton X-100 in PBS. The remaining nucleated cells were washed in PBS prior to fetal cell isolation.

#### Fetal cell isolation

Fetal cell enrichment and staining was performed using Magnetic Activated Cell Sorting (MACS®, Miltenyi Biotec, Bergisch Gladbach, Germany), as previously described [29]. The fetal cell–enriched fraction was applied to MagnetPick™ microscopic slides (Automated Lab Solutions, Jena, Germany), and cells were scanned and analyzed using an ALS-CellCelector™ (Automated Lab Solutions, Jena, Germany), with an in-house developed classifier software that identifies potential fetal cell candidates based on morphology and staining pattern. Representative images of stained fetal cells among maternal cells can be seen in the supplementary material and methods. The potential fetal cell candidates were subsequently manually validated according to ARCEDI’s established criteria, and one cell per tube was picked, when possible, followed by cell lysis [29]. In cases where two cells both passing fetal cell classification criteria were in physical contact with each other, they were collected together in one tube, as experience shows they are likely to be of the same origin and cannot be picked separately. In case no cells matched the criteria set for fetal cell classification, cells with a weaker match were isolated. A tube containing one or two collected cell(s) is referred to as a cell sample irrespective of the number of cells. Unless specified, cell samples contained a single cell. Lysates were stored at – 80 °C until shipped to the Department of Molecular Diagnostics, Aalborg University Hospital, and stored at − 20 °C.

#### Genetic testing of fetal cells

Genetic testing of DNA from fetal cells was performed as described above for PGT-M. Screening for aneuploidy was not performed during cbNIPT. In cases where the volume of the lysed cell samples differed from that of the embryo biopsy during PGT (all cases except case 8), the STR marker analysis and direct mutation detection setup previously used for PGT were tested in the new volume prior to analysis of the cell samples. Sequencing, data analysis, and visualizations were performed as described above for PGT-M. Failed tests due to either “no signal” and/or ADO could not be repeated as all fetal DNA was used in the initial reaction.

#### Classification of cell origin

An informative test result was defined as the ability to determine the origin of the cell sample as well as the mutational status (only relevant when of fetal origin). Classification of cell samples with respect to origin assumed that the pregnancy was a result of the transferred embryo following PGT-M and not a spontaneous conception. Isolated cells were classified as “fetal” if a paternal allele was detected. Detection of both maternal alleles for a given STR marker would result in a classification of the cell sample as “maternal,” as would the presence of only the maternal allele not inherited by the transferred embryo. The detection of only the maternal allele inherited by the transferred embryo would result in an “inconclusive” classification, as the origin of the cell could not be determined.

#### Classification of mutational status

STR markers linked to the unaffected allele are referred to as wildtype (wt) STR markers, while STR markers linked to the affected allele are referred to as mutant (mut) STR markers. Fetal cells were classified as “unaffected” in the case of (1) detection of at least one wt STR marker and absence of the mutation or (2) detection of at least two wt STR markers (one on each side of the mutation), when direct mutation detection was not possible or failed. In the absence of direct mutation detection, STR markers on both sides serve to ensure that the risk of a false negative only happens in the case of a double crossover event, which is considered extremely rare (for example, a 0.01% risk of an undetected crossover event if the two STR markers are located 1 Mb on each site of the mutation). When no direct mutation detection was performed and only one wt STR marker was present, the cell was classified as “conditionally unaffected,” and the estimated risk of crossover between the STR marker and the gene was provided. The risk of crossover was estimated based on the distance between the STR markers and the mutation sites in the genome, with 1 Mb distance given a 1% risk of crossover between the two sites.

### Chorionic villus sampling

CVS was performed at different hospitals depending on the patient’s place of residence. The CVS samples were subsequently sent to the Department of Clinical Genetics, Aarhus University Hospital, Denmark (cases 1–6 and 8) or the Molecular Genetics Laboratory, Department of Clinical Genetics, University Hospital Copenhagen, Denmark (case 7), for genetic analysis. The genetic analysis included mutation detection combined with a test for maternal contamination by STR marker analysis.

### Statistical analysis

Where appropriate, means ± standard deviations (SD) were calculated for normally distributed data while medians and range were calculated for data not normally distributed. Proportions were calculated and provided as percentages including exact Clopper-Pearson 95% confidence intervals (two-sided) where appropriate. All statistical analyses were performed using R version 3.6.1.

## Results

**Fig. 1:**
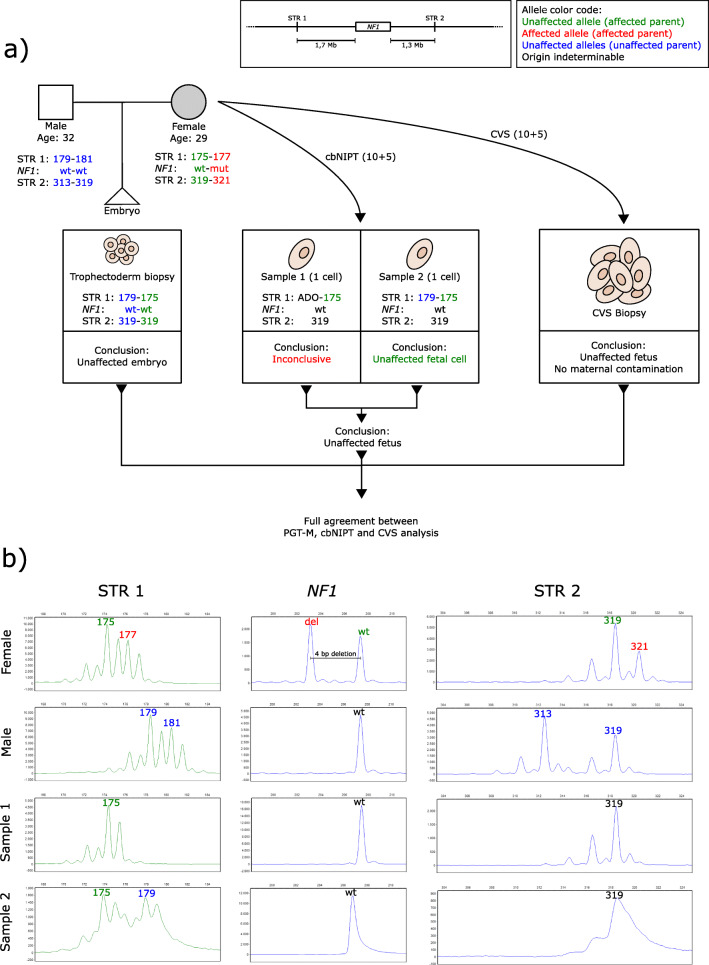
Results from case 1. **a** Flowchart describing the setup and STR markers used for PGT-M and cbNIPT as well as the results and conclusions from PGT, cbNIPT, and CVS. **b** STR profiles from cbNIPT including paternal and maternal profiles. Insert in the upper right corner details the affected gene and the locations of the STR markers used. Affected alleles are written in red, unaffected in green (the affected parent) or blue (the unaffected parent). Alleles of indeterminable origin are written in black. A couple (29 and 32 years old) seeking PGT due to the female partner being affected by neurofibromatosis type I caused by a deletion (c.7907+4_7del) in the *NF1* gene (**a**). Two STR markers, one fully informative located 1.7 Mb upstream of the *NF1* gene (STR 1, NF1-4, see supplementary materials and methods for genomic locations and sequence of self-annotated STR markers) and one semi-informative located 1.3 Mb downstream of the *NF1* gene (STR 2, D17S1880), were identified (**a** insert). Direct mutation detection (by fragment analysis because the mutation is a deletion) coupled with STR analysis was performed on DNA from lysed biopsied trophectoderm cells. An unaffected blastocyst was transferred resulting in pregnancy (**a**). CVS and blood sampling were performed in gestational week 10+5. Two potential fetal cell samples were isolated from the maternal blood sample (C1-S1 and C1-S2). C1-S2 was classified as inconclusive due to the absence of the paternal allele. C1-S2 was classified as an unaffected fetal cell with the same profile as the transferred embryo. Combined, cbNIPT confirmed the transfer of an unaffected embryo, which was also confirmed by CVS analysis (**a**). *bp*, base pair; *cbNIPT*, cell-based non-invasive prenatal testing; *CVS*, chorionic villous sampling; *Mb*, mega bases; *PGT*, preimplantation genetic testing; *PGT-M*, PGT for monogenic disorders; *STR*, short tandem, *Cx-Sy*, case x sample y

The characteristics of the eight cases are provided in Table 1. Mean female and male age at the time of gamete retrieval was 30.8 ± 3.5 and 32.5 ± 3.3, respectively. Eight different genes were affected, one gene in each case, of which six were of maternal origin. Two semi-informative or informative STR markers were identified and used for each case, except for cases 6 and 8 where three and one STR marker(s) were used, respectively. Mutation detection was used in seven cases, of which two were indirect, using STR markers located within the duplication (case 3) or deletion (case 6). In case 7, direct mutation detection was not possible as the unaffected maternal allele and both paternal alleles had the same number of repeats (non-informative). CVS and blood sampling were performed between gestational weeks 10+5 and 13+0. Figure 1 details the workflow and result from case 1. Detailed figures including STR profiles for the remaining seven cases are available in the supplementary material (Supplementary Figures 1, 2, 3, 4, 5, 6, 7).

**Fig. 2:**
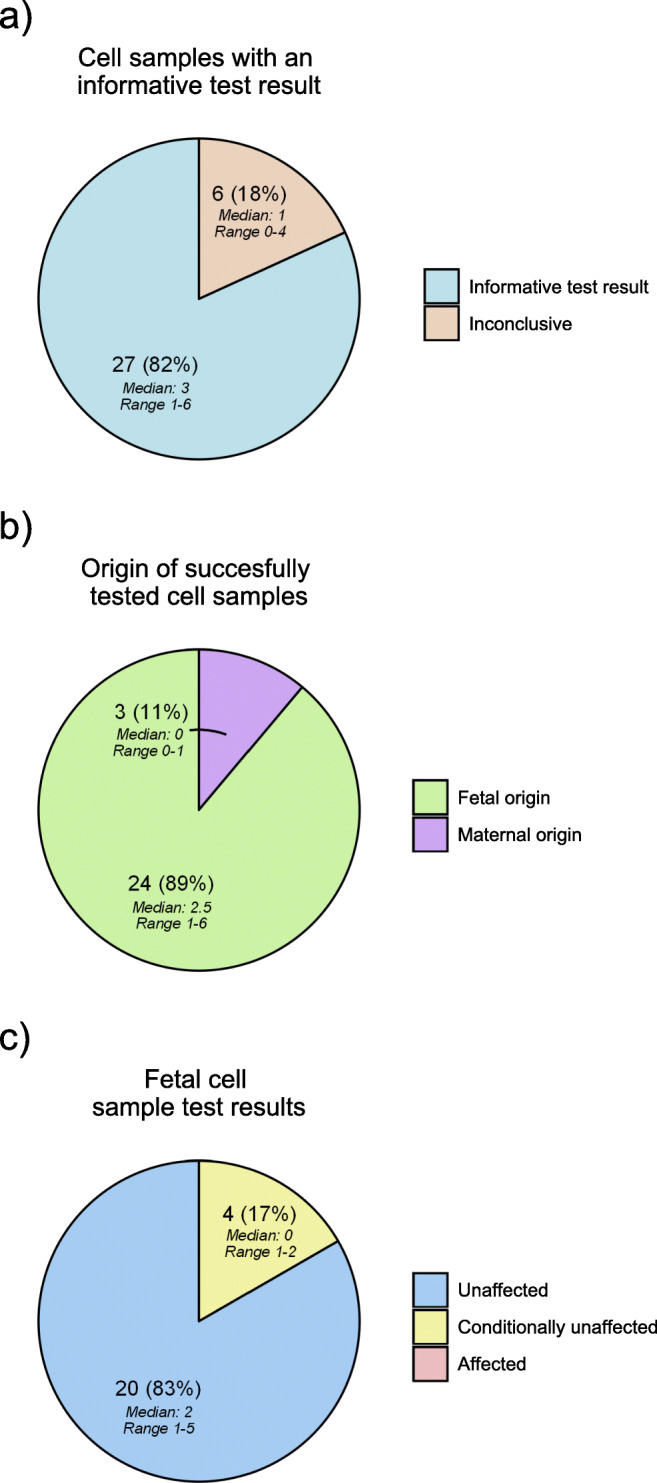
Piecharts of cbNIPT outcomes from the eight cases. **a** Proportion of cell samples succesfully tested. **b** Origin of successfully tested cell samples. **c** Proportion of fetal cell samples with an “unaffected,” “conditionally unaffected,” or “affected” test result. Number, percentage, range, and median are indicated on each slice of the pie charts

Results from cell isolation and cbNIPT are shown in Table 2 and Fig. 2. A total of 33 cell samples were collected with 27 (81.8%, CI_95_: 64.5–93.0%) having an informative test result (Fig. 2a). Per case, this corresponds to a median of 4.5 cell samples collected (range 2–6), of which a median of 3 cell samples had an informative test result (range 1–6, Fig. 2a). Six cell samples (18.2%, CI_95_: 7.0–35.5%) were inconclusive due to either no signal, ADO, or a combination, with ADO being the primary cause, corresponding to median of 1 per case (Fig. 2a). Of the STR marker tested, 55/66 (80.9%, CI_95_: 69.5–89.4%) produces a signal. Ninety-seven of 107 alleles were observed, resulting in an ADO rate of 9.4% (CI_95_: 4.6–16.5%).

**Table 2:** cbNIPT results

Case	Figure	Samples collected	Samples	Number of cells	Classification of cell origin	Test result	Percentage of cell samples successfully tested	CVS diagnosis	Concordance between cbNIPT and CVS
Case 1	Fig. 1	2	C1-S1	1	Inconclusive	-	50%	Unaffected	Yes
			C1-S2	1	Fetal cell	Unaffected			
Case 2	Supplementary figure 1	5^a^	C2-S1	1	No signal	-	60%	Unaffected	Yes
			C2-S2	1	Maternal cell	Unaffected			
			C2-S3	1	Inconclusive	-			
			C2-S4	1	Maternal cell	Unaffected			
			C2-S5	1	Fetal cell	Unaffected			
Case 3	Supplementary figure 2	5	C3-S1	1	Fetal cell	Unaffected	80%	Unaffected	Yes
			C3-S2	1	Fetal cell	Unaffected			
			C3-S3	1	No signal	-			
			C3-S4	1	Fetal cell	Unaffected			
			C3-S5	1	Fetal cell	Unaffected			
Case 4	Supplementary figure 3	2^a^	C4-S1	2	Maternal cell	Affected	50%	Unaffected	Yes
			C4-S2	1	Fetal cell	Unaffected			
Case 5	Supplementary figure 4	3	C5-S1	1	Fetal	Conditionally unaffected^b^	67%	Unaffected	Yes
			C5-S2	1	Fetal	Unaffected			
			C5-S3	1	Inconclusive	-			
Case 6	Supplementary figure 5	6	C6-S1	1	Fetal	Unaffected	100%	Unaffected	Yes
			C6-S2	1	Fetal	Unaffected			
			C6-S3	2	Fetal	Unaffected			
			C6-S4	2	Fetal	Conditionally unaffected ^c^			
			C6-S5	2	Fetal	Unaffected			
			C6-S6	1	Fetal	Unaffected			
Case 7	Supplementary figure 6	6	C7-S1	1	Fetal	Conditionally unaffected^d^	100%	Unaffected	Yes
			C7-S2	1	Fetal	Unaffected			
			C7-S3	1	Fetal	Unaffected			
			C7-S4	1	Fetal	Conditionally unaffected^e^			
			C7-S5	1	Fetal	Unaffected			
			C7-S6	2	Fetal	Unaffected			
Case 8	Supplementary figure 7	4^a^	C8-S1	1	No signal	-	75%	Unaffected	Yes
			C8-S2	2	Fetal	Unaffected			
			C8-S3	1	Fetal	Unaffected			
			C8-S4	2	Fetal	Unaffected			

^a^No cells fully matched the criteria set for fetal cell identification. Cells were selected based on a weaker classifier match

^b^Diagnosis based on STR 2 located 2.3 Mb downstream of the FMR1 gene, giving a risk of a false negative diagnosis of approximately 2.3% due to the risk of an undetected crossover event between STR 2 and FMR1

^c^Diagnosis based on STR 3 located 1.8 Mb downstream of the DMD gene, giving a risk of a false negative diagnosis of approximately 1.8% due to the risk of an undetected crossover event between STR 3 and DMD

^d^Diagnosis based on STR 1 located 1.9 Mb upstream of the DMPK gene, giving a risk of a false negative diagnosis of approximately 1.9% due to the risk of an undetected crossover event between STR 1 and DMPK

^e^Diagnosis based on STR 2 located 1.2 Mb downstream of the DMPK gene, giving a risk of a false negative diagnosis of approximately 1.2% due to the risk of an undetected crossover event between STR 1 and DMPK

*cbNIPT*, cell-based non-invasive prenatal testing; *CVS*, chorionic villous sampling; *Cx-Sy*, case x sample y

Of the 27 cell samples with an informative test result, 24 (88.9%, CI_95_: 70.8–97.7%) were of fetal origin, with the three remaining cell samples being of maternal origin (Fig. 2b). This corresponds to a median of 2.5 cell samples of fetal origin isolated per case (range 1–6, Fig. 2b). At least one cell sample of fetal origin was tested as unaffected (median 2, range 1–5) in all cases (Table 2, Fig. 1c). A median of 2.5 cell samples of fetal origin had either an unaffected or conditionally unaffected test result per case (range 1–6). Of the 24 fetal cell samples, 20 cell samples (83.3%, CI_95_: 62.6–95.2%) had an unaffected test result while 4 (16.7%, CI_95_: 4.7–37.4%) had a conditionally unaffected test result (Fig. 2c).

The majority of cell samples contained a single cell except for seven cell samples containing two cells (21.2%, CI_95_: 9.0–38.9%). In all seven cell samples, the STR profile revealed the two cells to be of the same origin (six fetal pairs and one maternal pair).

In three cases (cases 2, 4, and 8), none of the isolated cells fully matched the criteria set for fetal cell identification. Hence, cells with a weak match to the fetal classification criteria resulted in the isolation of 11 cell samples (one-third of all cell samples). Of these, five cell samples were subsequently shown to be of fetal origin (45.5%, CI_95_: 16.8–76.6%) and three of maternal origin (27.3%, CI_95_: 6.0–61.0%), while the remaining were inconclusive or produced no signal (Table 2). When only including cells that fully matched the fetal cell criteria (thus, excluding the cell samples in case 2, 4, and 8), 19/19 of cell samples with an informative test result were of fetal origin (100%, CI_95_: 82.4–100.0%).

## Discussion

The present case series indicates that cbNIPT is a promising future alternative to invasive testing by CVS when opting for prenatal confirmation of PGT-M. In all cases, the original PGT-M result was verified by cbNIPT and in agreement with the CVS analysis. Fetal cells were isolated with a specificity of 88.9% (CI_95_: 70.8–97.7%). Importantly, at least one fetal cell sample was successfully tested and gave an informative test result in all cases. An inconclusive test result was obtained in 18.2% (CI_95_: 7.0–35.5%) of cell samples due to no signal or ADO. ADO is expected when performing single-cell analysis, and the observed ADO rate of 9.4% (CI_95_: 4.6–16.5%) is similar to previously reported ADO rates [30, 31]. ADO is far less common when analyzing multiple cells, as is the case when analyzing trophectoderm cells (5–10 cells) during PGT-M. Hence, as a standard procedure of PGT, one STR marker on each side has been shown to be sufficient at our clinic. Optimizing the number of STR markers for single-cell cbNIPT might be needed to reduce the number of inconclusive test results due to ADO. The problem cannot be solved entirely by more STR markers, as increasing the complexity of the multiplex PCR might also increase the chance of ADO due to suboptimal individual PCR conditions when multiplexing. However, successful use of three or four STR markers during noninvasive prenatal single-cell diagnostics has previously been published [17, 25]. It should be noted that the special precautions described in a recent guideline for PGT laboratory procedures should be applied when testing single cells [32].

Depending on the estimated risk that an undetected crossover event has occurred, conditionally unaffected test results might require additional prenatal testing. Reducing ADO rates will probably move some of the cell samples out of the conditionally unaffected category, reiterating the need for testing and incorporating additional STR markers when performing cbNIPT compared to PGT. Another potential solution is to reduce the impact of ADO by increasing the number of isolated fetal cells available for testing, thereby increasing the chance of at least one cell having an informative test result. Importantly, no fetal cells analyzed by cbNIPT were discordant with the original PGT-M result and the genetic test of the CVS.

In this setting, all cells classified as fetal by the microscopy scanner were manually validated according to a specific set of criteria based on staining pattern and morphology [29]. In cases 2, 4, and 8, no cells were found to fully match the criteria for fetal cell identification. However, as no cells fully matched the criteria, five (case 2), two (case 4), and four (case 8) cells with weaker staining patterns were chosen for STR marker analysis (Table 2). Of the resulting 11 cell samples, eight were successfully tested, showing five and three to be of fetal and maternal origin, respectively. This indicates that not all fetal cells are identified with the current criteria, but loosening the criteria decreases the specificity as more maternal cells might be falsely identified as fetal cells during the isolation step. The specificity of fetal cell isolation could be increased to 100% (CI_95_: 82.4–100.0%) from 88.9% (CI_95_: 70.8–97.7%) when excluding cells that did not fully match the fetal cell classification criteria (cases 2, 3, and 5); however, the consequence would be that these three cases would have had no isolated cells for subsequent analysis (37.5% of all cases, CI_95_: 8.5–75.5%). Since cell origin is revealed by subsequent genetic analysis, thereby allowing maternal cells to be distinguished, loosening the criteria and thereby reducing the specificity might be a worthwhile trade-off to increase the number of fetal cells isolated, in order to further reduce the risk of not finding any fetal cells in a given case. This seems especially important considering the variation in the number of isolated fetal cells (range 1–6, Table 2, Fig. 1) between cases. Given the range of one to six fetal cells per case and 16.7% (CI_95_: 4.7–37.4%) of cell samples being conditionally unaffected, there is a considerable risk of cases with either no fetal cells or only fetal cells receiving an inconclusive or conditionally unaffected diagnosis, in which case additional testing would be needed. Increasing the number of isolated cells from the blood sample and/or reducing the number of inconclusive test results, e.g., by optimizing the number of STR markers, as mentioned previously, are possible solutions to this problem.

The only other report by Chang et al. [28] on cbNIPT following PGT-M included two couples, from which one and two candidate cells isolated from maternal blood were tested and confirmed to be fetal trophoblastic cells. Since the total number of isolated potential candidate cells was not reported and not all candidate cells analyzed, the specificity for fetal cell isolation cannot be estimated.

Following whole genome amplification (WGA) of the DNA, Chang et al. determined cellular origin using a commercial STR kit, while the mutational status was determined by PCR-based direct mutation detection. Additionally, they performed NGS-based analysis of single nucleotide polymorphisms (SNPs) as an alternative method to obtain information regarding the cellular origin and mutational status of the isolated cells by karyomapping. A downside of karyomapping is that direct mutation detection is not always possible. Hence, at least in some cases, a separate analysis for direct mutation detection has to be performed. However, a benefit of karyomapping, which utilizes SNPs as genetic markers, is the relative high prevalence of SNPs compared to STR markers, which means that multiple informative SNPs are more easily found and often in closer vicinity to the gene of interest.

As stated by the authors, WGA on single cells is associated with amplification failure and high ADO rates, which might affect the reliability of downstream analysis. Our observed ADO rate of 9.4% (CI_95_: 4.6–16.5%) when performing PCR directly on the isolated cells was comparable to or lower than the 26.7% when performing SNP analysis following WGA and the 18.8% (6/32) using a commercial STR kit reported by Chang et al. However, WGA allows for multiple lines of analysis and concurrent aneuploidy screening if warranted.

We present a single PCR-based setup without the need for WGA consisting of STR marker analysis of custom-designed STR markers in close vicinity to the gene of interest coupled with direct mutation detection capable of determining cellular origin and mutational status. Our setup has the additional benefit of being cheaper than WGA and NGS-based solutions, which can be an important factor, especially in countries where the cost of cbNIPT is to be covered by the patient and therefore might affect decision making.

Placental mosaicism, the presence of multiple genetically distinct cells lines, can pose a challenge during prenatal testing as the genetic status of the embryo is inferred from testing placental cells. As placental mosaicism arises from mitosis during embryonic development, it has no impact when testing for hereditary disorders but might complicate interpretation when screening for aneuploidies. Since placental cells are used for both CVS, cffDNA, and cbNIPT, the issues associated with genetic testing on placental cells and inferring the result to the embryo remains independent of the method, and the current clinical guidelines for CVS should be applied in cases when aneuploidy and/or mosaicism is detected.

Despite the existence of numerous commercial solutions related to single-cell isolation and analysis [24], widespread clinical implementation of cbNIPT is likely awaiting evidence demonstrating a robustness comparable to that of CVS. Hence, invasive testing is still the gold standard. Considering that prenatal testing is sometimes declined due to its invasive nature and associated risks, cbNIPT might not only provide a more acceptable alternative from a patient’s perspective, but it may also increase the percentage of pregnancies receiving prenatal testing [5–7]. This would increase the chance of detecting a potential PGT misdiagnosis prenatally. Hence, efforts should be made to move cbNIPT into clinical practice. Importantly, since fetal cells have been isolated as early as gestational week 5 [17], cbNIPT could potentially be performed early in pregnancy, allowing CVS to be performed within the limit of legal abortion in many countries (including Denmark, where the limit is gestational week 12) in the case of an inconclusive cbNIPT result. If this method were to be implemented into clinical practice, blood samples would have to be sent to a laboratory trained in the proprietary method developed by ARCEDI Biotech, which will then return individual isolated cells for genetic testing. This has the advantage that the requesting laboratory or clinic can choose their own method of genetic testing, as in our example where the original PGT-M setup could be applied reducing both time to answer and cost of the test. A drawback might be that ARCEDI Biotech is currently only located in Denmark but given that fetal cells can be isolated up to 48 h after blood sampling, this solution should be possible for most clinics around the world.

The data provided here are promising with respect to a suitable alternative to invasive testing following PGT-M in the future. However, given the small dataset, more cases are needed to properly evaluate the robustness of the procedure prior to clinical implementation.

## Conclusion

Here we present eight cases where cbNIPT was used to confirm pregnancy with an unaffected fetus following PGT-M. There was concordance between cbNIPT, PGT, and CVS results for all cases. Fetal cells were isolated with high specificity. Informative test results were obtained for all cases. ADO was the primary reason for inconclusive cell samples warranting an optimization of STR marker analysis for cbNIPT. Fetal cells were still identified when loosening the fetal cell criteria, indicating this as a way to increase the number of isolated fetal cells. ARCEDI Biotech is continuously working on improving the procedure to increasing the number of isolated fetal cells. At the time of publication, patient enrollment is still ongoing and the accumulation of more data will allow us to better evaluate cbNIPT as a prenatal test following PGT-M and how it might benefit patients. The results presented here illustrate the potential of cbNIPT as an alternative to invasive testing following PGT-M.

## Supplementary Information

ESM 1 (DOCX 2.05 mb)
