# Supplementary material for: Cell-based non-invasive prenatal testing for monogenic disorders: confirmation of unaffected fetuses following preimplantation genetic testing
Source: J Assist Reprod Genet. 2021 Mar 7;38(8):1959–70. doi: 10.1007/s10815-021-02104-5 (PMC8417213; doi:10.1007/s10815-021-02104-5)
Supplement: Supplementary file 1 — (DOCX 2.05 mb) [file 10815_2021_2104_MOESM1_ESM.docx]

# Supplementary material

## Supplementary materials and methods

### In vitro fertilization, embryo culture, biopsy and transfer

Patients underwent ovarian stimulation in a short antagonist protocol (Ganirelix) using urinary (Menopur^©^, Ferring Pharmaceuticals, Saint-Prex, Switzerland) or recombinant FSH (Gonal-F^©^, Merck Serono, Darmstadt, Germany). FSH doses were adjusted individually according to the patients’ ovarian response. A dose of 10,000 IU of hCG (Ovitrelle^©^, Merck Serono, Darmstadt, Germany) or in the case of risk of ovarian hyperstimulation syndrome, 0,2 mg Gonapeptyl^©^ (Ferring Pharmaceuticals, Saint-Prex, Switzerland) was administered when at least three follicles measured 17 mm or more by ultrasound monitoring. Oocyte retrieval was conducted using ultrasound-guided puncture of ovarian follicles 36 hours later. Retrieved oocytes were denudated and placed in Cook fertilization media (Cook Medical, Bloomington, United States of America).

Intracellular sperm injection was performed on all metaphase 2 oocytes, and the injected oocytes were transferred individually into microwells of EmbryoSlide culture dishes (Vitrolife, Göteborg, Sweden) pre-equilibrated with cleavage media (Cook Medical, Bloomington, United States of America) overlayered with oil (Origio, Måløv, Denmark) and cultured in a time lapse incubator (EmbryoScope^©^, Vitrolife, Göteborg, Sweden) at 37 °C, 6.0 % CO_2_ and 5 % O_2_ .

The cleavage media was exchanged with blastocyst media (Cook Medical, Bloomington, United States of America) two days post fertilization, and the embryos were further cultured to the blastocyst stage, which was reached 5- or 6-days post fertilization depending on the developmental kinetics of the developing blastocyst.

The blastocysts were graded according to Gardner and Schoolcraft [1]. Expanding or expanded blastocysts with or without herniating cells and a grade of at least BB underwent trophectoderm biopsy as previously described [2].

Approximately five to ten trophectoderm cells were biopsied per embryo. Biopsied trophectoderm cells were washed in biopsy medium (Origio, Måløv, Denmark), lysed by shock freezing in liquid nitrogen, treated with 2.5 µl protease K (Roche, Basal, Switzerland), overlaid with 25 µl oil (Origio, Måløv, Denmark) and shipped on dry ice to the genetic laboratory and stored at -20 °C. The blastocysts were vitrified using vitrification media (Kitazato, Tokyo, Japan) immediately following biopsy and stored in liquid nitrogen at -196 °C awaiting the result of genetic testing of the biopsies. Single unaffected blastocysts were transferred into the uterus in subsequent frozen embryo transfer (FET) treatment(s) either in a substituted or modified natural cycle.

Pregnancy was defined as the presence of a fetal heartbeat monitored by ultrasound in gestational week 7.

## Preimplantation genetic testing

STR markers were tested using the AmpliTaq Gold™ DNA polymerase (Applied biosystems, Thermo Fisher Scientific, Waltham, Massachusetts, United States of America) according to the manufacturer’s instructions and customized fluorescently labeled primers (https://www.ncbi.nlm.nih.gov/tools/primer-blast/) (in a total volume of 12.5 µl). When STR markers were detected in a multiplex reaction alongside a repeat expansion mutation, AmpliTaq Gold® 360 DNA Polymerase (Applied biosystems, Thermo Fisher Scientific, Waltham, Massachusetts, United States of America) was used according to the manufacturer’s instructions.

### Direct mutation detection

Direct mutation detection was performed when possible to further increase diagnostic accuracy. Primer sequences can be found in the supplementary materials and methods in Table S1. The different types of mutations and their detection methods are detailed below.

#### Smaller deletions

Smaller deletions were tested using customized fluorescent labelled primers and the same kit as during STR marker analysis (described above), thus allowing for a distinction between the mutant and wild type based on the size of the PCR product. Smaller gene deletions were tested in a multiplex PCR reaction alongside the STR markers.

#### Point mutations

To detect point mutations, mini sequencing was performed using fluorescent labelled nucleotides and the SNaPshot® multiplex Kit (Applied biosystems, Thermo Fisher Scientific, Waltham, Massachusetts, United States of America) according to the manufacturer’s instructions. Primers were placed on each site of the point mutation so that only the nucleotide at the site of the mutation was to be incorporated during the PCR reaction. Different fluorophores coupled to each of the four bases allowed for a distinction between the wild type and mutant.

#### Repeat expansion

In the case of repeat expansions, the extended allele may not be detected due to its increased length. Hence, a diagnosis is based on the presence or absence of the wildtype allele of the affected parent. In the case of repeat expansion mutations, STR markers and the repeat expansion were detected using the AmpliTaq Gold® 360 DNA Polymerase (Applied biosystems, Thermo Fisher Scientific, Waltham, Massachusetts, United States of America) according to the manufacturer’s instructions.

#### Larger duplications and deletions

Larger duplications and deletions were detected by the absence or presence of STR markers located within the deleted or duplicated region. Hence, detection was performed as described for STR marker analysis.

### Sequencing and fragment analysis and data analysis

All PCR products were analyzed on an ABI 3500 genetic analyzer (Applied biosystems, Thermo Fisher Scientific, Waltham, Massachusetts, United States of America). Data analysis and visualization was performed using GeneMarker® version 2.6.4 (Softgenetics®, State College, United States of America).

### Images of stained fetal cell

Below are examples of stained fetal cells (green and blue) surrounded by maternal cells (only blue). Blue color represents the cell nucleus while green color represents fetal cell specific markers.


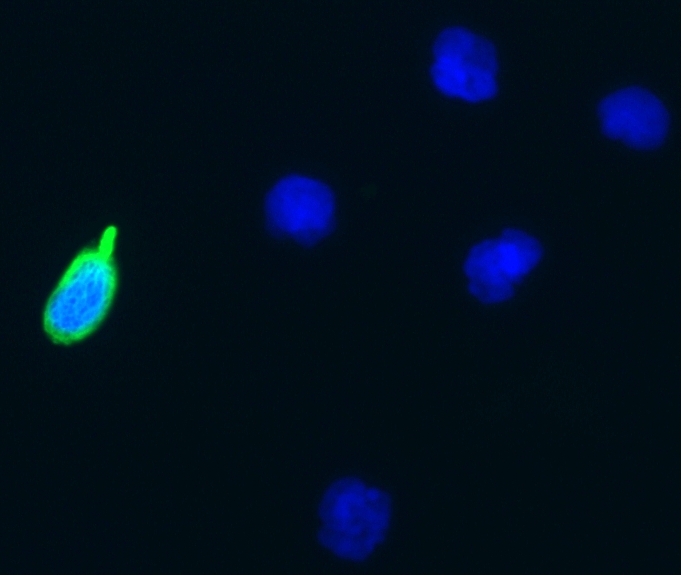


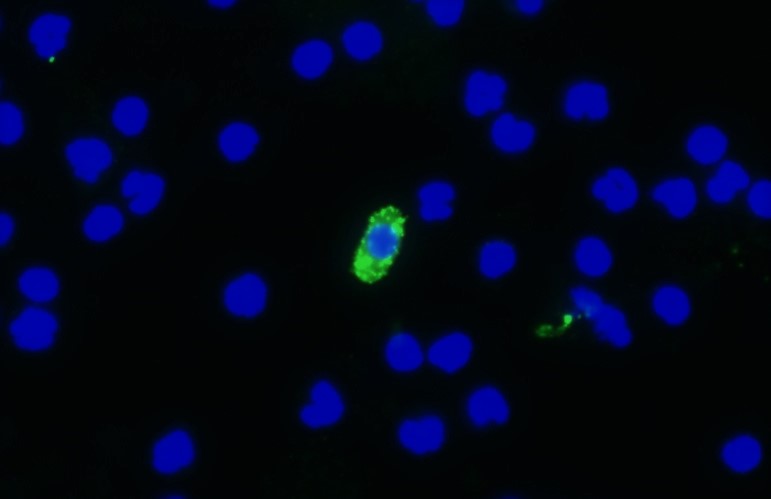


### STRs and primers

Primer sequences are provided in table S1 below.

| Table S1: Primer sequences | | | | |
| --- | --- | --- | --- | --- |
| Case | STR/ gene | Locus ID/mutation | Forward primer (5’-3´)  (x denotes position of Hex probe) | Reverse primer (5’-3´) |
| 1 | STR 1 | NF1-4* | xTTCTGCTCCTTGGGCCCCTGT | AGGGATAAGCTGTACAGTATGGGGAC |
|  | *NF1* | c.7907+4_7del | xTCTTGGCAGGCTACACTGGT | TCATTTTGCCCTCTTTGCAACC |
|  | STR 2 | D17S1880 | xACCCTGTTTCAAAGCGGGAGGG | TGGCCCTTGCCTGTGGTACT |
| 2 | STR 1 | D2S2255 | xCCTGGGTCTCCAGGCTACTT | AGCTTGCTTGTCTCAGGTCA |
|  | *SPG4* | c.481delG | xAGCAAAATAGGACTCACGGC | CTGAAATCTGGACAATCATGTGAA |
|  | STR 2 | SPG4-2* | xGCAAACTGTGAGGATTTCGGC | GATCAGCCCTCCATATCCAGT |
| 3 | STR 1 | D17S122 | xGGCCCTATTTCACTCCCAGT | TTGGCATTTTTGGTGGCAGG |
|  | STR 2 | D17S900 | xTGAAGTACCCAGTATCAATCAGGT | GTCTGTCAGCAGCTTGCTTT |
| 4 | STR 1 | D19S892 | xCTAGGCTGGAGATCTGGGAAC | CTCATTCCTAGACACGCCCTG |
|  | *NOTCH3* | c.520T>C | CGGGTGGGTGAGCCCTGCCGCCATGGTGGCACC | GACACTGGCAGCGGAAGGAGCCAGGTGTGTTGAGGC |
|  | STR 2 | D19S252 | xCAGTGGACCCAATTAACATTCCAA | TGGGTTACCTGTTCGTTCTCT |
| 5 | *FMR1* | FRAXA1* | xGCTCAGCTCCGTTTCGGTTTCACTCCCGGT | AGCCCCGCACTTCCACCACCAGCTCCTCCA |
|  | STR 1 | DXS1193 | xCCCAATTCTGACTCTGGGGCCT | TCCAGCAGGGTGGCTGAGACAA |
|  | STR 2 | DXS8086 | xATATACATTGCATTCCCTCTGGGCCT | CCAGCTTGCTGACTCACCCTG |
| 6 | STR 1 | DXS8039 | xAAACTAACAAACCTCTAGCCAG | TTCATGTCCATGTGAACAG |
|  | STR 2 | DXS997 | xTGGCTTTATTTTAAGAGGAC | GTTTTCAGTTTCCTGGGT |
|  | STR 3 | DMD67* | xACATCTCCTGTTCCCCAAAACTATT | TCCACCTGTTTCCACCCTTGACACA |
| 7 | STR 1 | D19S538 | xTGCAGAGCCTAGAGCCTGGAGT | GCGTGAACGGGGCGTGTCAT |
|  | STR 2 | D19S545 | xTCTGGTGGAGATTTCCAGATGCTGT | GGGAGGCTGAGGTGGGAAGACT |
| 8 | *FBN1* | c.1148-2A>G | xAGGATGACTTCTGTGGGCCT | ATTTTTGAATTCTTACTTGGTGGCT |
|  | STR 1 | D15S978 | xAGCTTCATACACTGAAATTGTTG | CACCGGGAAACCTTGAT |
| *Newly identified STRs  Abbreviations: STR: Short Tandem Repeat | | | | |

DNA sequences for STR markers including up- and downstream DNA sequence are provided below. Identified STR markers are marked with yellow. Primers are marked with green (corresponding to the sequences provided in Table S1).

### NF1-4:

STR: CT-repeat

Genomic position (hg38): chr17:31062627-31063576

Sequence:

AATGACAGGCTGGCCAATCAGATGCTCTCACCCTAGACTTTATATTGTGA

ATAAAAGTGTGGTGTGAGGCAAAGATGAATGGAAAGCTAGAATCCATGTG

CATTGGCTGGGTGTGGTGGCTCACGCCTGTAATCCCAGCACTCTGGGAGG

CCGAGGCTGGCAGATCACCTGAGGTCAGGAGTTCGAGACCATCCCGGCCA

ACAAGGTGAAACCCTGTCTCTACTAAAAATACAAAAATTAACTGGGCATG

GTGGCATGAGCCTGTAATCCCAGCTACCCAGGAGGCTGAGGCAGGAGAAT

CTCTGGAACCCAGGAGGCAGAGGCTACAGTGAGCTGAGATTGCGCCACTG

CACCCCAGCCTGGGTGACAGGGCAAGACTCCATCTCAAAAAAAAAAAAAA

AAGAATCCATGTGCATTGGAAGGATCTGGCCAAGGCATGCTGGTAGGTTT

TTTCCTGCTCAAAGATGATTATCTTGTATTCTGCTCCTTGGGCCCCTGTC

TCTATTGGTTTTTGGCCCATATAGTTCTCTCTCTCTCTCTCTCTCTCTCT

CTCTCTCTCTCTCTCTTTCTCTCTCTGTGTGTGTGTGTTTGATGGCAAAT

TTCAAATATACAGTACATTATTATCAATCATAGTCCCCATACTGTACAGC

TTATCCCTATCGTCATTCTTCACCTTCTAATAATGTTTGTTGCTTCTTAC

ATATTTTGAAGCCTCATAGAAACTTGAAACTATTTAATTCAAAGTGGCCT

CTTAGTCTCTTTTCTGCTTACGATAATCGGAGTGGTTTTCTGCTGCTTGC

AACCAACAATTGACTATATAGACATGTTTGGGAGGCATAATATAATGCTG

ATTTTTTAGGTGTTGTAGGGATACCAAGATGAATGCAGTCTGGAATGCAC

TACACACAGCGCATGAGAATACGGGCTCCGTAGTGAGACAGGCTTCAGTT

### SPG4-2:

STR: TA-repeat

Genomic position (hg38): chr2:33256325-33257178

ATTCTCCCCTTCAGAAACACTTCCCCTTTTCCGTCCTCCGTAATCTACAT

CCTCTTAACCAGCTGATACATGGCATTTTGGTGGAGCCCTTTGTGTTCTT

CAACTGGAAGCAAACTGTGAGGATTTCGGCATCTCATTTCCCTGAATAGT

GATTTTTTTTAACCTTATGGCTCTTCATCTAACTTAGACAAATGTCACTG

AGTTCTTTTTTAATAACTAATGAAATTACATCAAAGCGGGAAAAAAAGCC

TGATTTTTGCTGATAAGACATATACATGCAACATGAGATATATAATACAT

ATAGTGTCTTCTATATATAAAATATCTCCTAATATATAACCTAATATGAT

ATATAGTATTTTCTAATTGGTTGCTGATATATATGGGATGGAGGGCTAAC

TATATATATATATATATATATATATATATATATATATATATATATATATA

TATAGTTACCCTCTGTATCCATGGTTATGCATCAGTGGATTCAACCCAAT

TGGTTGGTTGAATCTGTGGATGCAGAACTGGATATGGAGGGCTGATCATA

CTATGCAATTTTATGTAAGGGTCTTGAGCATTGCAGATTTTGATATCCAC

AGGGGTTATCCTAGAACTAATCCCTCATGGATACTGAGGAATGACTATGT

AAATAATATGATATATAAAATATATGCTAAGTATATGCTATATATAATAT

GTATATTCTGATTTGGGTAGGATAAGGAATTAACAGATGGAAAAATGTTT

AGTAAAAAAAAAGTTGGATTTTTTTCTTTAAATATGCAAGTTTATCAGAA

ATTCGAGCAATTTGCTTACATTTTGCACCTGTTTTTGAAGTTTTAAAATG

TAAC

### FRAXA1

STR: CGG-repeat

Genomic position (hg38): chrX:147911730-147912410

Sequence:

CGCCCGGCCCGCGCGTCTGTCTTTCGACCCGGCACCCCGGCCGGTTCCCA

GCAGCGCGCATGCGCGCGCTCCCAGGCCACTTGAAGAGAGAGGGCGGGGC

CGAGGGGCTGAGCCCGCGGGGGGAGGGAACAGCGTTGATCACGTGACGTG

GTTTCAGTGTTTACACCCGCAGCGGGCCGGGGGTTCGGCCTCAGTCAGGC

GCTCAGCTCCGTTTCGGTTTCACTTCCGGTGGAGGGCCGCCTCTGAGCGG

GCGGCGGGCCGACGGCGAGCGCGGGCGGCGGCGGTGACGGAGGCGCCGCT

GCCAGGGGGCGTGCGGCAGCGCGGCGGCGGCGGCGGCGGCGGCGGCGGCG

GAGGCGGCGGCGGCGGCGGCGGCGGCGGCGGCTGGGCCTCGAGCGCCCGC

AGCCCACCTCTCGGGGGCGGGCTCCCGGCGCTAGCAGGGCTGAAGAGAAG

ATGGAGGAGCTGGTGGTGGAAGTGCGGGGCTCCAATGGCGCTTTCTACAA

GGTACTTGGCTCTAGGGCAGGCCCCATCTTCGCCCTTCCTTCCCTCCCTT

TTCTTCTTGGTGTCGGCGGGAGGCAGGCCCGGGGCCCTCTTCCCGAGCAC

CGCGCCTGGGTGCCAGGGCACGCTCGGCGGGATGTTGTTGGGAGGGAAGG

ACTGGACTTGGGGCCTGTTGGAAGCCCCTCT

### DMD67:

STR: GAAA- and GA-repeat

Genomic position (hg38): chrX:33504000-33504539

Sequence:

AGCTAAGGAATAAGAATGCAAAGGCATAAGAATGATATAATAGACTTTGG

GAACTCAAGGGAAAGGCTGAGAGAGGGGTGAGGGATAAAAGACTGCACAT

TGGGTACAGTGTACACTGCTCAGGTGATGAGTGTACCAAAATCTCAGAAA

TCACCATTAAAGAACTTACCCATGTAACTAAACATCTCCTGTTCCCCAAA

ACTATTGAAATACTGAAAGAAAGAAAGAAAGAAAGAAAGAGAGAGAGAGA

GAGAGAGAGAGAAAGAAAGAAAGAAAGAAAGAAAGAAAGAAAGAAAGAAA

GAAAGAAAGAAAGAAAAAGAAAGAAGGAAGGAAAGAAATTCTTTAATAAG

ACTTTGATATGGTTTGGCTGTGTCCCCACCCAAAATCTTATCTTGATTTG

TAATCTCCCAAATCTCCATGATCCCCATGTGTCAAGGGTGGAAACAGGTG

GAGTTACTTGGATCATGGAGAGGGTTTTGCACATCCTGTTCTTGTGATAA

TGAGTGGGTCTCAAAAGATCTGATGGTTTTATAAGCATCT

# References:

1. Gardner DK, Schoolcraft WB (1999) In vitro culture of human blastocysts. In: Jansen R, Mortimer D (eds) Toward Reproductive Certainty: Fertility and Genetics Beyond 1999. London: Parthenon Publishing, pp 378–388

2. Capalbo A, Rienzi L, Cimadomo D, et al (2014) Correlation between standard blastocyst morphology, euploidy and implantation: An observational study in two centers involving 956 screened blastocysts. Hum Reprod 29:1173–1181. https://doi.org/10.1093/humrep/deu033

## Supplementary results

### Supplementary Figure 1


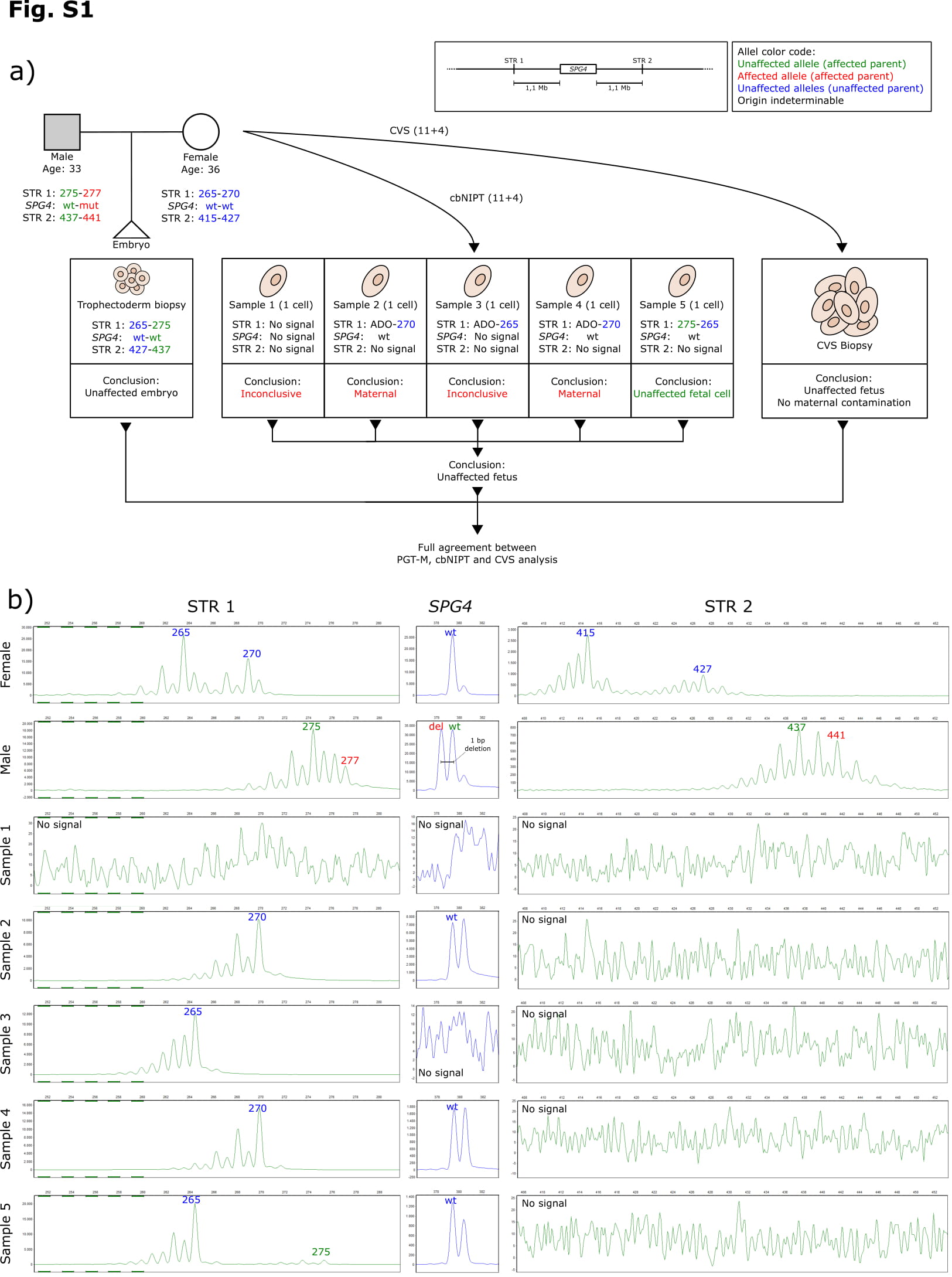


### Supplementary Figure 1 legend

Results from case two. a) Flowchart describing the setup, process and STR markers used for PGT and cbNIPT as well as the results and conclusions from PGT and cbNIPT. b) STR profiles from cbNIPT including paternal and maternal profiles. Insert in the upper right corner details the affected gene and the locations of the STR markers used. Affected alleles are written in red, unaffected in green (the affected parent) or blue (the unaffected parents). Alleles of indeterminable origin are written in black.

Case two involved a couple seeking PGT due to the male partner being affected by spastic paraplegia type 4, caused by a deletion (c.481delG) in the *SPG4* gene. The female and male partner were 36 and 33 years old, respectively, at the time of gamete retrieval and egg fertilization. Two fully informative STR markers located 1.1 Mb upstream (STR 1, D2S2255) and downstream (STR 2, D17S1880) of the *SPG4* gene were identified. Direct mutation detection (fragment analysis due to mutation being a deletion) coupled with STR analysis were performed on DNA from lysed biopsied trophectoderm cells. An unaffected blastocyst was transferred, resulting in pregnancy. CVS and blood sampling were performed in gestational week 11+4. No cells in the blood sample passed the full fetal cell criteria as set by ARCEDI Biotech, but 5 cells with a weaker staining pattern were identified and isolated as possible fetal cells from the maternal blood sample (C2-S1 through C2-S5). C2-S1 and C2-S3 were inconclusive as the analysis either gave no signal (C2-S1) or did not allow us to determine the origin of the cell due to absence of paternal STR markers (C2-S3). C2-S2 and C2-S4 were classified as maternal cells, as the maternal STR marker not inherited by the transferred embryo was detected. C2-S5, despite there being no signal for STR 2, was classified as an unaffected fetal cell due to the presence of the unaffected paternal allele (STR 1) and the absence of the *SPG4* c.481 deletion, corresponding to the profile of the transferred embryo. Combined, cbNIPT confirmed the transfer of an unaffected embryo, which was also confirmed by CVS analysis.

Abbreviations: bp: base pair; cbNIPT: cell-based non-invasive prenatal testing; CVS: chorionic villous sampling; Mb: mega bases; PGT: preimplantation genetic testing; PGT-M: PGT for monogenic disorders; STR: short tandem repeat; Cx-Sy: Case x, sample y

### Supplementary Figure 2


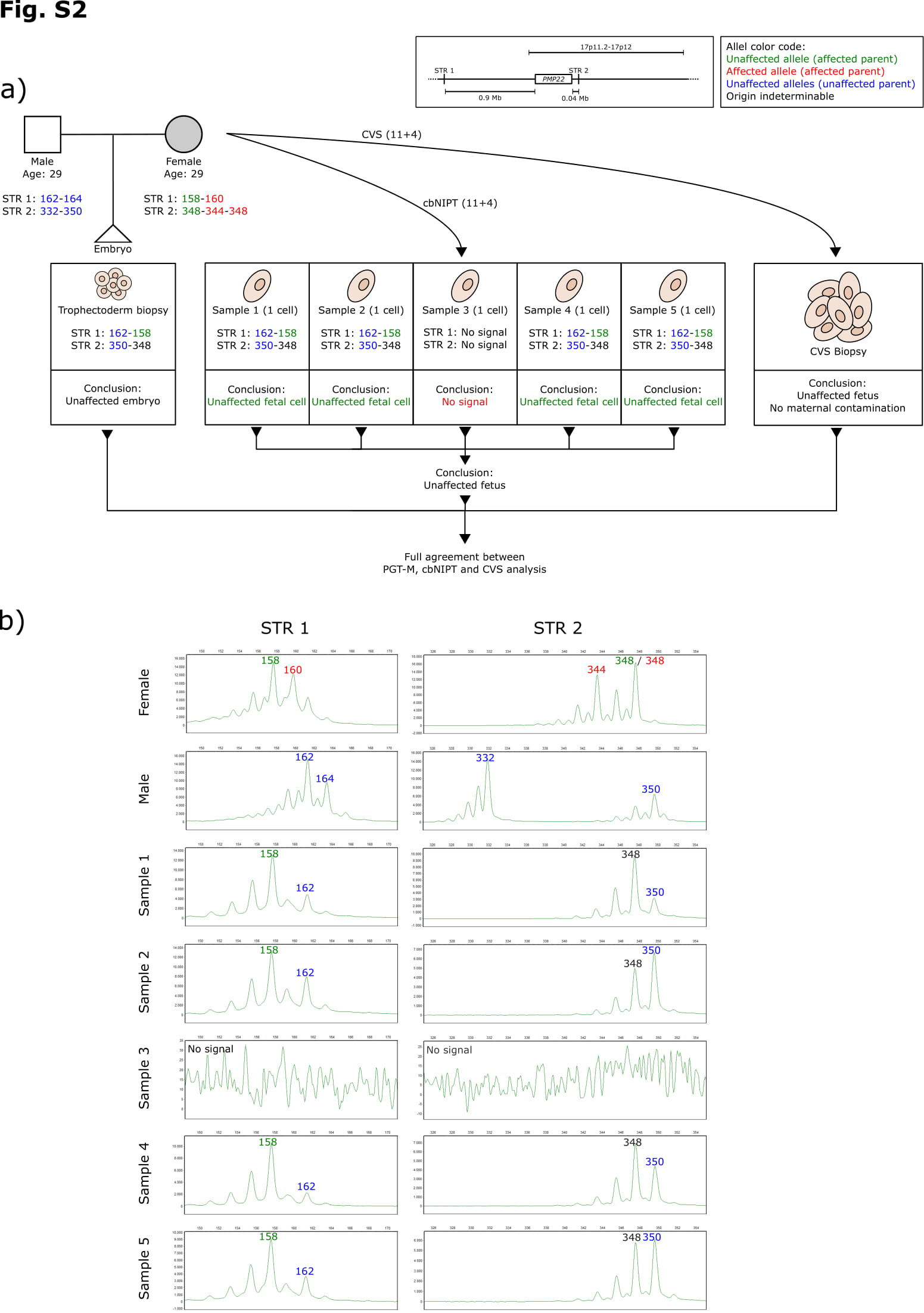


### Supplementary Figure 2 legend

Results from case three. a) Flowchart describing the setup, process and STR markers used for PGT and cbNIPT as well as the results and conclusions from PGT and cbNIPT. b) STR profiles from cbNIPT including paternal and maternal profiles. Insert in the upper right corner details the affected gene and the locations of the STR markers used. Affected alleles are written in red, unaffected in green (the affected parent) or blue (the unaffected parents). Alleles of indeterminable origin are written in black.

Case three involved a couple seeking PGT due to the female being affected by Charcot Marie Tooth Type A (CMT1A) caused by a duplication of p11.2p12 on chromosome 17, (p11.2p12)dup(17), containing the *PMP22* gene. The female and male partner were both 29 years old at the time of gamete retrieval and egg fertilization. An informative STR marker located 0.9 Mb upstream (STR 1, D17S122) and a semi-informative STR marker located 0.04 Mb downstream (STR 2, D17S900) of the *PMP22* gene were identified. STR 1 and STR 2 were located outside and inside the duplicated region, respectively. STR marker analysis was performed on DNA from lysed biopsied trophectoderm cells. An unaffected blastocyst was transferred resulting in pregnancy. CVS and blood sampling were performed in gestational week 11+4. Five potential fetal cell samples were isolated from the maternal blood sample (C3-S1 through C3-S5). Four cells were classified as unaffected fetal cells (C3-S1, C3-S2, C3-S4 and C3-S5) while one cell did not produce any signal (C3-S3). The STR profiles of the four cell samples all matched the profile of the transferred embryos. Combined, cbNIPT confirmed the transfer of an unaffected embryo, which was also confirmed by CVS analysis.

Abbreviations: bp: base pair; cbNIPT: cell-based non-invasive prenatal testing; CVS: chorionic villous sampling; Mb: mega bases; PGT: preimplantation genetic testing; PGT-M: PGT for monogenic disorders; STR: short tandem repeat; Cx-Sy: Case x, sample y

### Supplementary Figure 3


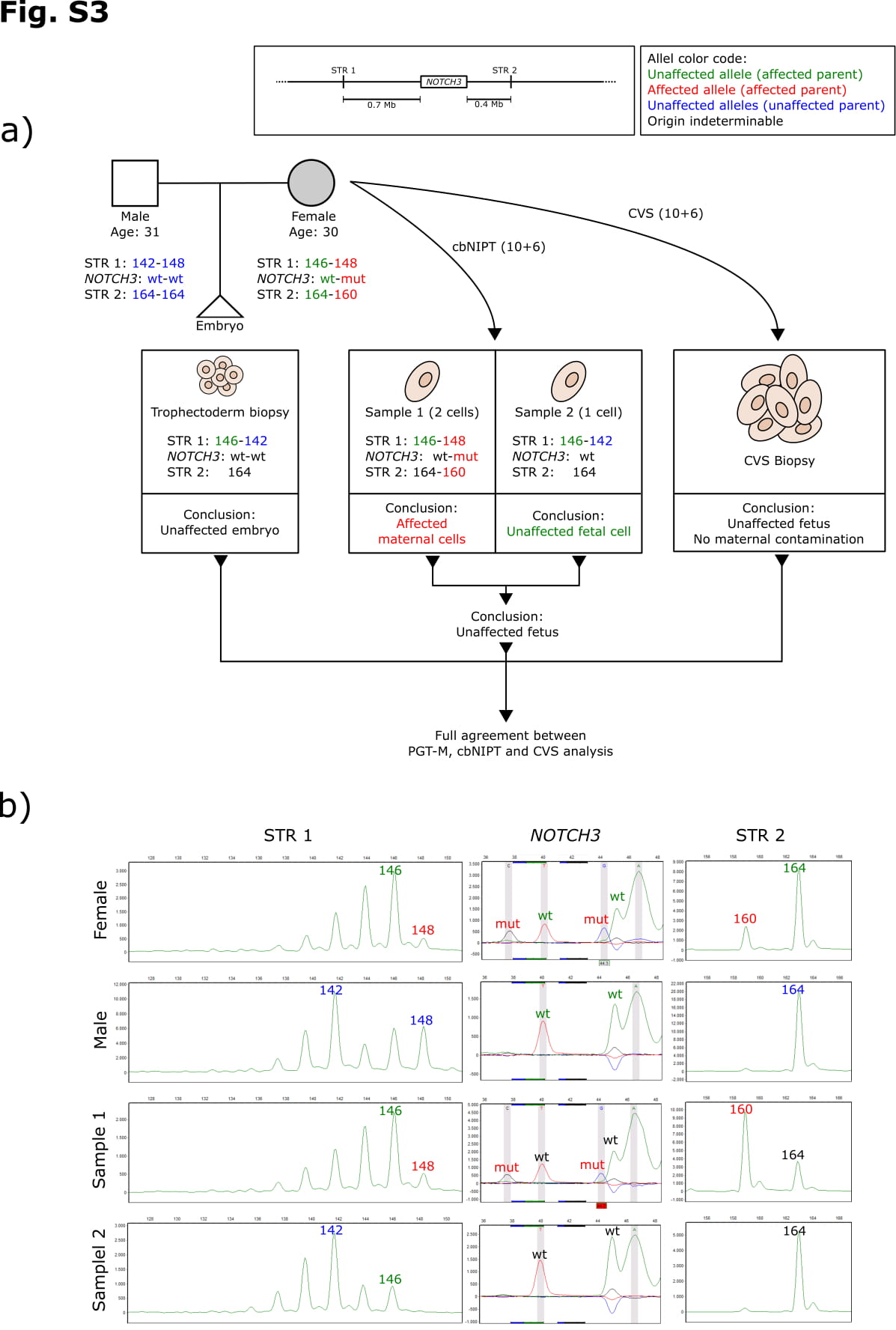


### Supplementary Figure 3 legend

Results from case four. a) Flowchart describing the setup, process and STR markers used for PGT and cbNIPT as well as the results and conclusions from PGT and cbNIPT. b) STR profiles from cbNIPT including paternal and maternal profiles. Insert in the upper right corner details the affected gene and the locations of the STR markers used. Affected alleles are written in red, unaffected in green (the affected parent) or blue (the unaffected parents). Alleles of indeterminable origin are written in black.

Case four involved a couple referred to PGT due to the female being affected by Cerebral autosomal dominant arteriopathy with subcortical infarcts and leukoencephalopathy (CADASIL) caused by a point mutation (c.520T>C) in the *NOTCH3* gene. The female and male partner were 30 and 31 years old, respectively, at the time of gamete retrieval and egg fertilization. Two semi-informative STR markers were identified located 0.7 Mb upstream (STR 1, D19S892) and 0.4 Mb downstream (STR 2, D19S252) of *NOTCH3*. Direct mutation detection coupled with STR marker analysis was performed on DNA from lysed biopsied trophectoderm cells. An unaffected blastocyst was transferred resulting in pregnancy. CVS and blood sampling were performed in gestational week 10+6. No cells in the blood sample passed the full fetal cell criteria as set by ARCEDI Biotech, but two potential fetal cell samples with a weaker staining pattern were isolated (C4-S1 and C4-S2). One sample contained two cells (C4-S1). C4-S2 was classified as an unaffected fetal cell showing the same profile as the transferred embryo. Analysis of C4-S1 showed no paternal markers, two maternal markers for STR 1 and the affected maternal STR 2 allele coupled with detection of the mutation, indicating an affected maternal cells. Combined, cbNIPT confirmed the transfer of an unaffected embryo, which was also confirmed by CVS analysis.

Abbreviations: bp: base pair; cbNIPT: cell-based non-invasive prenatal testing; CVS: chorionic villous sampling; Mb: mega bases; PGT: preimplantation genetic testing; PGT-M: PGT for monogenic disorders; STR: short tandem repeat; Cx-Sy: Case x, sample y

### Supplementary Figure 4


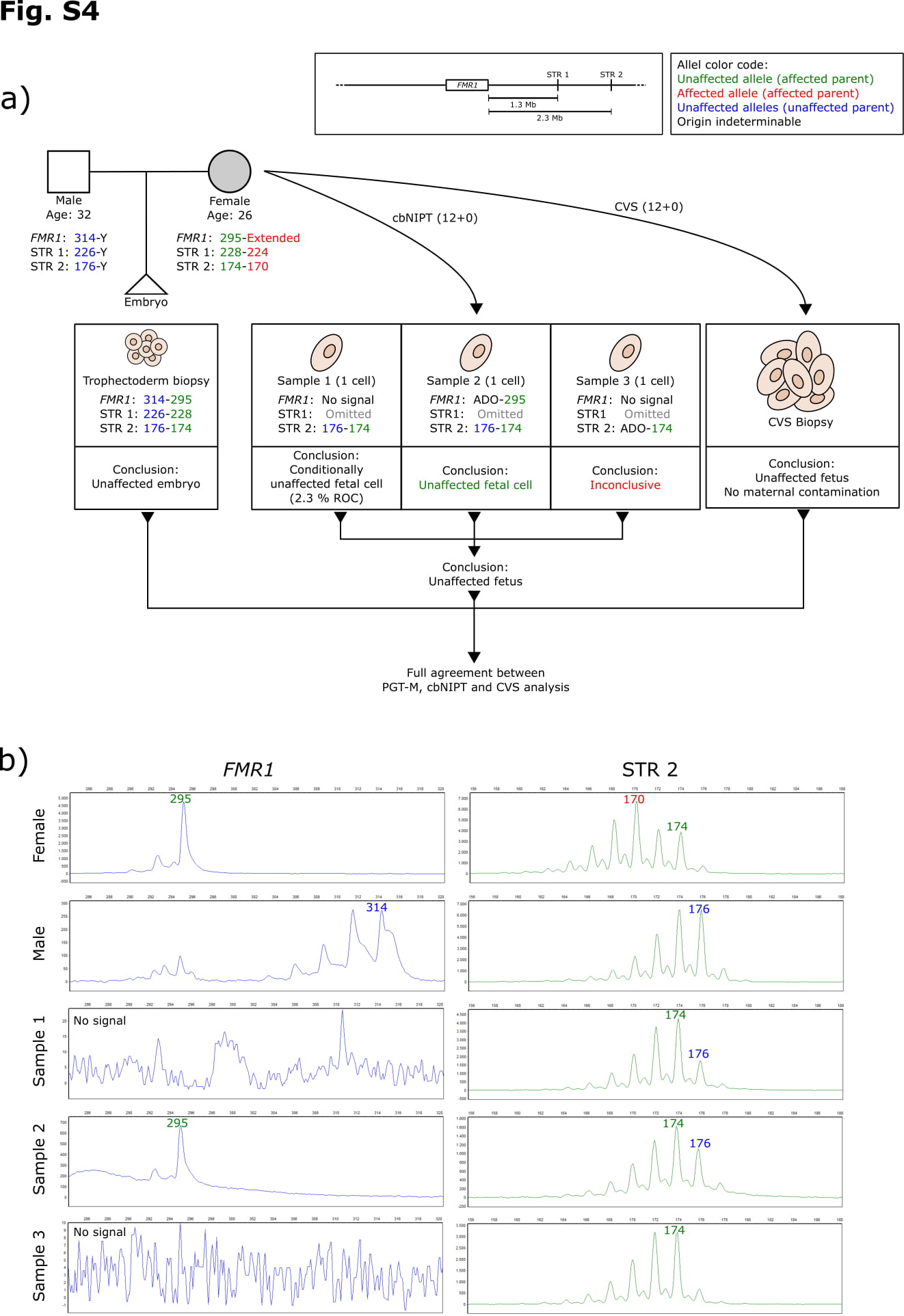


### Supplementary Figure 4 legend

Results from case five. a) Flowchart describing the setup, process and STR markers used for PGT and cbNIPT as well as the results and conclusions from PGT and cbNIPT. b) STR profiles from cbNIPT including paternal and maternal profiles. Insert in the upper right corner details the affected gene and the locations of the STR markers used. Affected alleles are written in red, unaffected in green (the affected parent) or blue (the unaffected parents). Alleles of indeterminable origin are written in black.

Case five involved a couple seeking PGT due to the female partner being having Fragile X syndrome caused by a CGG-repeat expansion on one allele of the *FMR1* gene. The female and male partner were 26 and 32 years old, respectively, at the time of gamete retrieval and egg fertilization. No informative STR markers were identified upstream of the *FMR1* gene. Two fully informative STR markers located 1.3 (STR 1, DXS1193) and 2.3 (STR 2, DXS8086) Mb downstream of the *FMR1* gene were identified. Direct mutation detection (fragment analysis due to the nature of the mutation) coupled with STR marker analysis were performed on DNA from lysed biopsied trophectoderm cells. An unaffected blastocyst was transferred resulting in pregnancy. CVS and blood sampling were performed in gestational week 12+0. Three potential fetal cell samples were isolated from the maternal blood sample (C5-S1 through C5-S3). STR 2 did not produce a signal when tested in the double volume used for single cell analysis. Hence, it was omitted from the single cell analysis. C5-S1 showed both paternal and maternal markers but no signal for *FMR1*. Hence, C5-S1 was classified as a conditionally unaffected fetal cell with approximately 2.3 % risk of a false negative diagnosis due to an undetected crossover event. C5-S2 was classified as an unaffected fetal cell since it showed both maternal and paternal markers along with the wildtype maternal *FMR1* allele. C5-S3 produced no signal for *FMR1* and no paternal markers were detected resulting in an inconclusive diagnosis. Combined, cbNIPT confirmed the transfer of an unaffected embryo, which was also confirmed by CVS analysis.

Abbreviations: bp: base pair; cbNIPT: cell-based non-invasive prenatal testing; CVS: chorionic villous sampling; Mb: mega bases; PGT: preimplantation genetic testing; PGT-M: PGT for monogenic disorders; STR: short tandem repeat; Cx-Sy: Case x, sample y

### Supplementary Figure 5


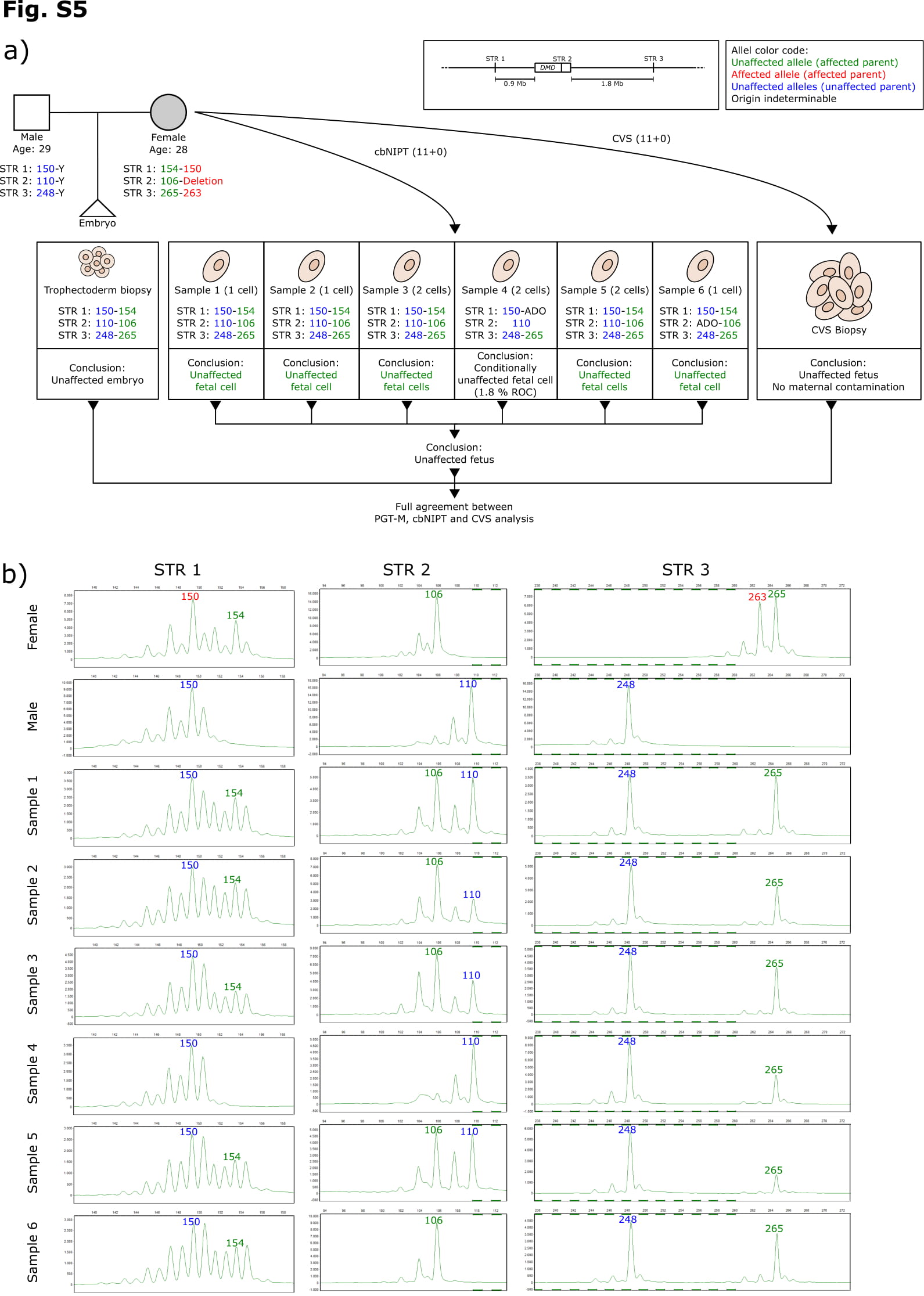


### Supplementary Figure 5 legend

Results from case six. a) Flowchart describing the setup, process and STR markers used for PGT and cbNIPT as well as the results and conclusions from PGT and cbNIPT. b) STR profiles from cbNIPT including paternal and maternal profiles. Insert in the upper right corner details the affected gene and the locations of the STR markers used. Affected alleles are written in red, unaffected in green (the affected parent) or blue (the unaffected parents). Alleles of indeterminable origin are written in black.

Case six involved a couple seeking PGT due to the female partner being affected by Duchenne and Becker muscular dystrophy due to a deletion of exon 47 and 48 of the *DMD* gene. The female and male partner were 28 and 29 years old, respectively, at the time of gamete retrieval and egg fertilization. One semi-informative STR marker located 0.9 Mb upstream of the *DMD* gene (STR 1, DXS8039) and two fully informative STR markers located within the deletion (STR 2, DXS997) and 1.8 Mb downstream (STR 3, DMD67, see supplementary materials and methods for genomic locations and sequence of self-annotated STR markers) of the *DMD* gene were identified. STR 2 located within the deletion served as direct mutation detection. STR marker analysis was performed on DNA from lysed biopsied trophectoderm cells. An unaffected blastocyst was transferred resulting in pregnancy. CVS and blood sampling were performed in gestational week 11+0. Six potential fetal cell samples were isolated from the maternal blood sample (C6-S1 through C6-S6). Three sample contained two cells (C6-S3, C6-S4 and C6-S5). All samples except C6-S4 were classified as unaffected fetal cells as they showed both maternal and paternal markers along with the wildtype maternal *DMD* allele. C6-S4 showed no maternal markers for STR 1 and STR 2, the latter of which serves as the direct mutation detection. Hence, C6-S4 was classified as a conditionally unaffected fetal cell with approximately 1.8 % risk of a false negative diagnosis due to an undetected crossover event. Combined, cbNIPT confirmed the transfer of an unaffected embryo, which was also confirmed by CVS analysis.

Abbreviations: bp: base pair; cbNIPT: cell-based non-invasive prenatal testing; CVS: chorionic villous sampling; Mb: mega bases; PGT: preimplantation genetic testing; PGT-M: PGT for monogenic disorders; STR: short tandem repeat; Cx-Sy: Case x, sample y

### Supplementary Figure 6


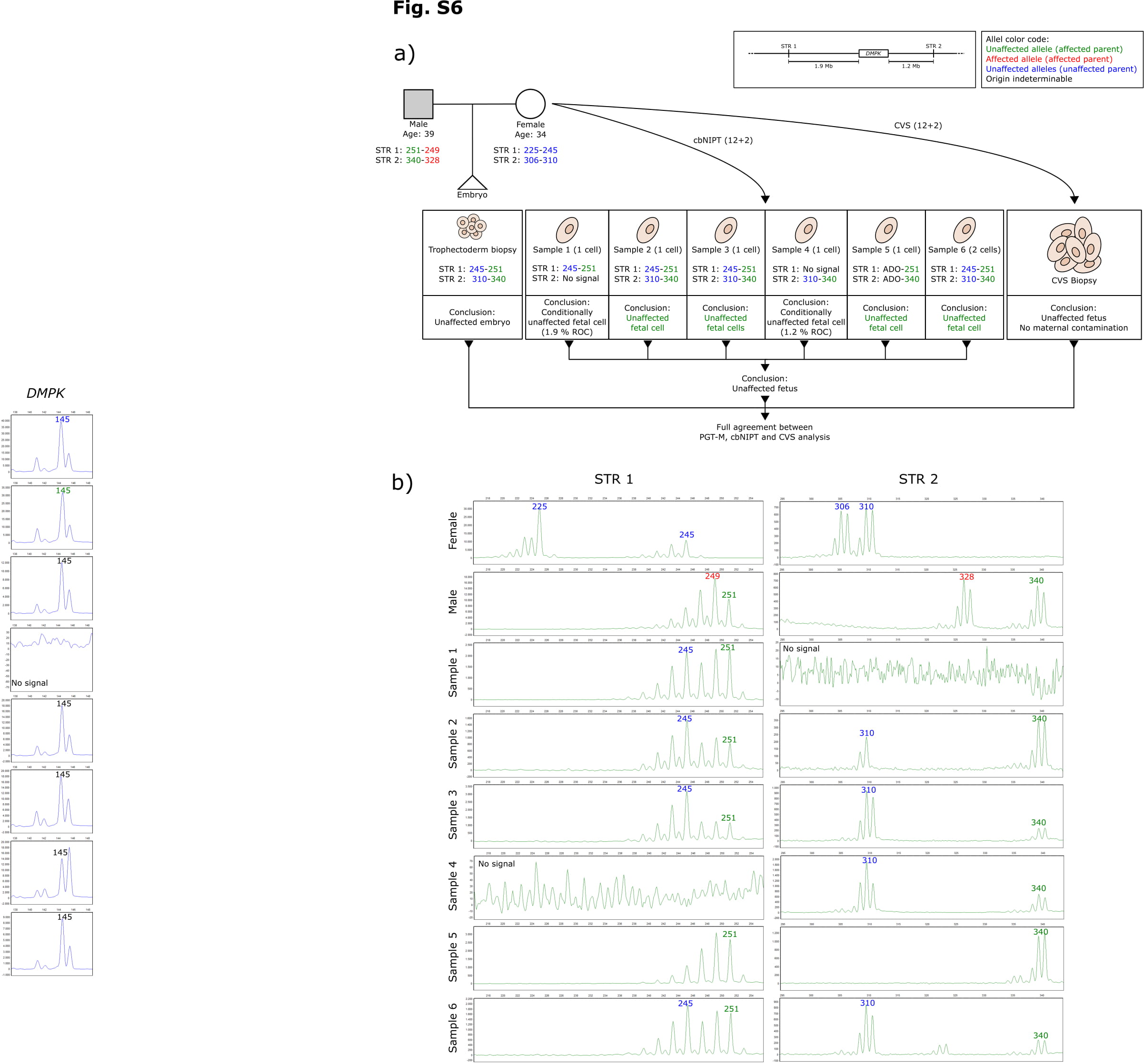


### Supplementary Figure 6 legend

Results from case seven. a) Flowchart describing the setup, process and STR markers used for PGT and cbNIPT as well as the results and conclusions from PGT and cbNIPT. b) STR profiles from cbNIPT including paternal and maternal profiles. Insert in the upper right corner details the affected gene and the locations of the STR markers used. Affected alleles are written in red, unaffected in green (the affected parent) or blue (the unaffected parents). Alleles of indeterminable origin are written in black.

Case seven involved a couple seeking PGT due to the male partner being affected by Myotonic dystrophy type 1 due to a CTG-repeat expansion within the *DMPK* gene. The female and male partner were 34 and 39 years old, respectively, at the time of gamete retrieval and egg fertilization. two fully informative STR markers located 1.9 Mb upstream (STR 1, D19S538) and 1.2 Mb downstream (STR 2, D19S545) of the *DMPK* gene were identified. Direct mutation detection was not possible since the couple shared an identical number of CTG-repeats between both maternal alleles and the wildtype paternal allele. STR marker analysis was performed on DNA from lysed biopsied trophectoderm cells. An unaffected blastocyst was transferred resulting in pregnancy. CVS and blood sampling were performed in gestational week 12+2. Six potential fetal cell samples were isolated from the maternal blood sample (C7-S1 through C7-S6). One sample contained two cells (C7-S6). Samples C7-S2, C7-S3, C7-S5 and C7-S6 were classified as unaffected fetal cells. Samples C7-S1 and C7-S4 were classified as conditionally unaffected fetal cells with approximately 1.9 and 1.2 % risk of a false negative diagnosis, respectively, due to an undetected crossover event, as there were no signal for STR 2 nor STR 1, respectively. Combined, cbNIPT confirmed the transfer of an unaffected embryo, which was also confirmed by CVS analysis.

Abbreviations: bp: base pair; cbNIPT: cell-based non-invasive prenatal testing; CVS: chorionic villous sampling; Mb: mega bases; PGT: preimplantation genetic testing; PGT-M: PGT for monogenic disorders; STR: short tandem repeat; Cx-Sy: Case x, sample y

### Supplementary Figure 7


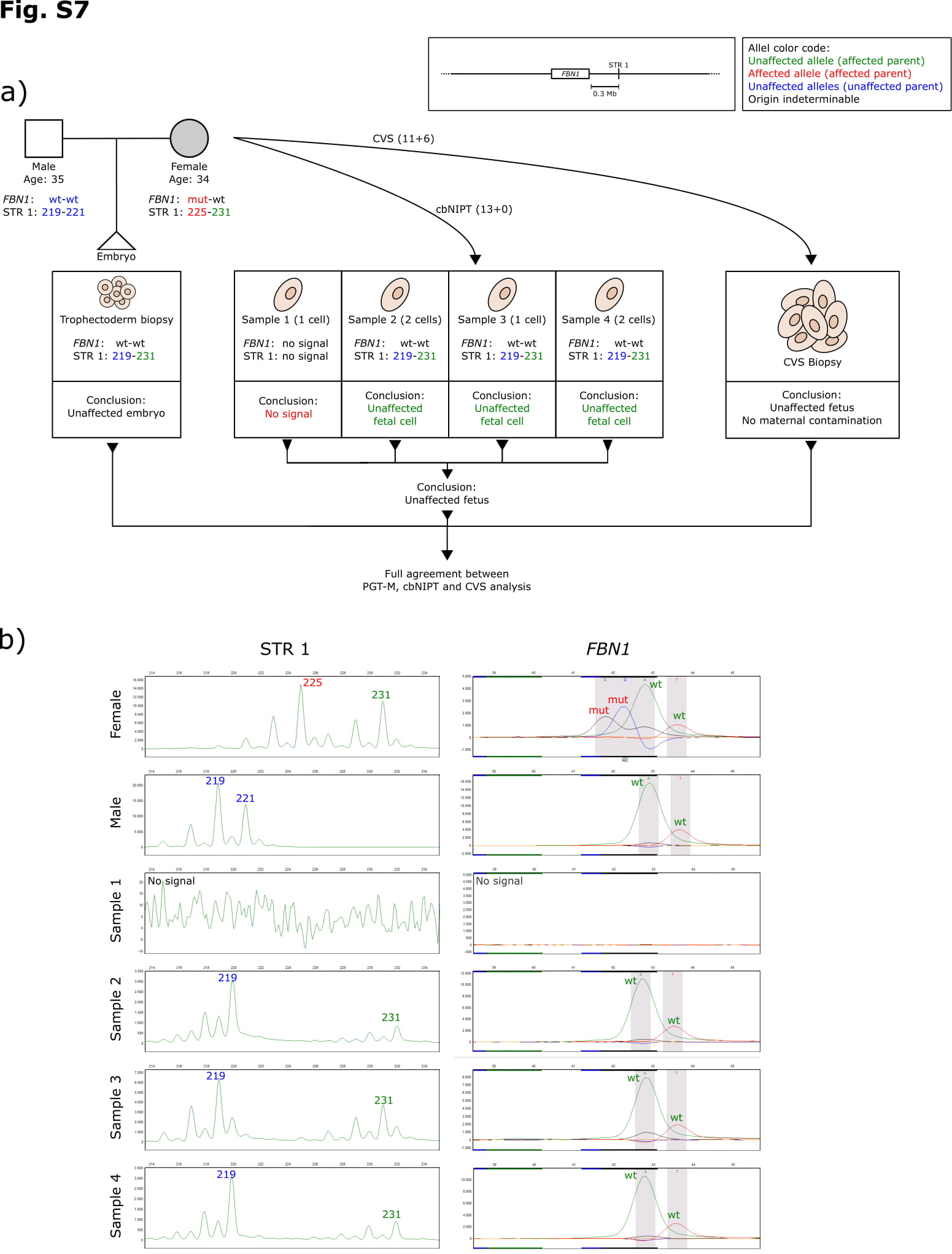


### Supplementary Figure 7 legend

Results from case eight. a) Flowchart describing the setup, process and STR markers used for PGT and cbNIPT as well as the results and conclusions from PGT and cbNIPT. b) STR profiles from cbNIPT including paternal and maternal profiles. Insert in the upper right corner details the affected gene and the locations of the STR markers used. Affected alleles are written in red, unaffected in green (the affected parent) or blue (the unaffected parents). Alleles of indeterminable origin are written in black.

Case eight involved a couple seeking PGT due to the female being affected by Marfan syndrome caused by a splice site mutation c.1148-2A>G in the *FBN1* gene. The female and male partner were 34 and 35 years old at the time of gamete retrieval and egg fertilization, respectively. A single informative STR marker located 0.3 Mb downstream (STR 1, D15S978) of the *FBN1* gene were identified. STR marker analysis was performed on DNA from lysed biopsied trophectoderm cells. An unaffected blastocyst was transferred resulting in pregnancy. CVS and blood sampling were performed in gestational week 11+6 and 13+0, respectively. Four potential fetal cell samples were isolated from the maternal blood sample (C3-S1 through C3-S5). Three cells were classified as unaffected fetal cells (C8-S2, C8-S3 and C8-S4) while one cell did not produce any signal (C8-S1). The STR profiles of the three embryos all matched the profile of the transferred embryo. Combined, cbNIPT confirmed the transfer of an unaffected embryo, which was also confirmed by CVS analysis.

Abbreviations: bp: base pair; cbNIPT: cell-based non-invasive prenatal testing; CVS: chorionic villous sampling; Mb: mega bases; PGT: preimplantation genetic testing; PGT-M: PGT for monogenic disorders; STR: short tandem repeat; Cx-Sy: Case x, sample y
